# Glial cell reactivity and oxidative stress prevention in Alzheimer’s disease mice model by an optimized NMDA receptor antagonist

**DOI:** 10.1038/s41598-022-22963-x

**Published:** 2022-10-25

**Authors:** Júlia Companys-Alemany, Andreea L. Turcu, Santiago Vázquez, Mercè Pallàs, Christian Griñán-Ferré

**Affiliations:** 1 Pharmacology Section, Department of Pharmacology, Toxicology, and Therapeutic Chemistry, Faculty of Pharmacy and Food Sciences, Institut de Neurociències, Universitat de Barcelona (NeuroUB), Av. Joan XXIII 27-31, 08028 Barcelona, Spain; 2 Laboratory of Medicinal Chemistry (CSIC Associated Unit), Department of Pharmacology, Toxicology, and Therapeutic Chemistry, Faculty of Pharmacy and Food Sciences and Institute of Biomedicine (IBUB), University of Barcelona, Av. Joan XXIII, 27-31, 08028 Barcelona, Spain

**Keywords:** Drug discovery, Molecular biology, Neuroscience

## Abstract

In Alzheimer's disease pathology, several neuronal processes are dysregulated by excitotoxicity including neuroinflammation and oxidative stress (OS). New therapeutic agents capable of modulating such processes are needed to foster neuroprotection. Here, the effect of an optimised NMDA receptor antagonist, UB-ALT-EV and memantine, as a gold standard, have been evaluated in 5XFAD mice. Following treatment with UB-ALT-EV, nor memantine, changes in the calcineurin (CaN)/NFAT pathway were detected. UB-ALT-EV increased neurotropic factors (*Bdnf*, *Vgf* and *Ngf*) gene expression. Treatments reduced astrocytic and microglial reactivity as revealed by glial fibrillary acidic protein (GFAP) and ionized calcium-binding adapter molecule 1 (Iba-1) quantification. Interestingly, only UB-ALT-EV was able to reduce gene expression of *Trem2*, a marker of microglial activation and NF-κB. Pro-inflammatory cytokines *Il-1β*, *Ifn-γ, Ccl2* and *Ccl3* were down-regulated in UB-ALT-EV-treated mice but not in memantine-treated mice. Interestingly, the anti-inflammatory markers of the M2-migroglial phenotype, *chitinase-like 3* (*Ym1*) and *Arginase-1* *(Arg1),* were up-regulated after treatment with UB-ALT-EV. Since *iNOS* gene expression decreased after UB-ALT-EV treatment, a qPCR array containing 84 OS-related genes was performed. We found changes in *Il-19*, *Il-22*, *Gpx6*, *Ncf1*, *Aox1* and *Vim* gene expression after UB-ALT-EV. Hence, our results reveal a robust effect on neuroinflammation and OS processes after UB-ALT-EV treatment, surpassing the memantine effect in 5XFAD.

## Introduction

*N*-Methyl-D-Aspartate receptors (NMDARs) and aberrant post-synaptic calcium signalling have been implicated in neurodegenerative conditions, including Alzheimer's Disease (AD)^1^. However, the physiological activity of NMDARs is necessary to mediate some aspects of development, synaptic transmission and normal neuronal function^2,3^. In AD, high levels of glutamate can be released from glial cells favouring excitotoxicity and leading to a massive influx of Ca^2+^ mediated by NMDARs^4^, promoting cell death^5^, oxidative stress (OS)^6^, and neuroinflammation^7^. Thus, the activation of several inflammatory signalling pathways promotes the synthesis of many proinflammatory mediators, including cytokines as well as other immune mediators such as reactive oxygen species (ROS), fostering a chronic neuroinflammatory state triggered by glial reactivity (especially microglia and astrocytes)^7,8^.

Considering these facts, it is important to note that inflammatory response, glial activation and OS are three associated mechanisms that emerge as main factors for AD progression^9^. Pursuing this vicious cycle between ROS and glial activation, the inflammatory regulatory signalling molecules, which are directly activated by calcineurin (CaN), nuclear factor of activated T-cells (NFAT) and the nuclear factor kappa-light-chain-enhancer of activated B cell (NF-κB), can be modulated by ROS increase^10–12^. Thus, in AD, both signalling pathways can feed a forward cycle in which cytokine production increases amyloid-β (Aβ) and neurofibrillary tangles (NFTs) production that mediates the release of more cytokines and maintain a continuous environment of inflammation and gliosis^13^. Moreover, a balance between ROS production and the antioxidant defence is essential for neuronal synaptic functionality^14^; when this equilibrium is inadequate, the accumulation of ROS triggers a deleterious response in the brain^15^. In AD, there is an unbalanced activity of the antioxidant enzymatic machinery^16^. Indeed, a link between ROS and neuroinflammation has been widely described via nitric oxide synthase (iNOS)^17^. iNOS activity and expression are tightly regulated by cytokines. Therefore, inflammatory process control directly impacts OS, resulting in a deleterious effect on neuronal functionality^18^.

For decades AD treatment has included memantine, a non-competitive NMDAR antagonist, combined with acetylcholinesterase inhibitors^19^. Although these drugs’ effectiveness has been seriously questioned in Alzheimer's patients in advanced stages^20,21^, they are the only clinical option available, as the efficacy of the recently amyloid β-directed monoclonal antibody aducanumab is still controversial^22,23^. It is well-known that memantine reduces neuronal damage and ameliorates memory and learning dysfunction^24,25^. All these beneficial effects could induce a reduction in neuroinflammation and consequently a cerebral function improvement. Furthermore, several pathways modulated by NMDAR blockade have been implicated in neuroprotection against excitotoxicity, in addition to those directly activated by calcium dysregulation^26^. All these beneficial effects could induce a reduction of neuroinflammation and OS, and consequently a cerebral function improvement, reinforcing the idea of targeting NMDARs to reduce neuroinflammation in AD. Nevertheless, as mentioned, clinical trials have shown that memantine displays a lack of effectivity in severe AD^27^. Therefore, new NMDAR antagonists with a higher capability to modulate glutamate signalling are needed.

We have developed an optimized NMDAR antagonist, UB-ALT-EV, with an excellent compromise between potency as an NMDAR channel blocker and druggable properties^28^. Strikingly, the compound UB-ALT-EV exhibits better neuroprotective effects than memantine in 5XFAD mice^28^. The 5XFAD transgenic mouse model is well-established for describing the molecular alterations in AD associated with Aβ accumulation, co-expressing five familial AD mutations^29^. Importantly, 5XFAD also showed an association between cognitive impairment, chronic neuroinflammation and OS markers suggesting the contribution of glial activation and OS in AD pathology^29^.

In the present study, we delved into the modulation of signalling pathways exerted by UB-ALT-EV compared to memantine, focusing on glial reactivity, neuroinflammatory response, and OS by using the 5XFAD mouse model. Therefore, we evaluated several inflammatory pathways, including their cytokines, as well as we determined changes in gene expression of OS markers related to the triggering of neuroinflammation following memantine and UB-ALT-EV treatments in 5XFAD mice.

## Results

### NMDAR antagonist treatments modified CaN/NFATc1 signaling pathway in 5XFAD mice

**Figure 1:**
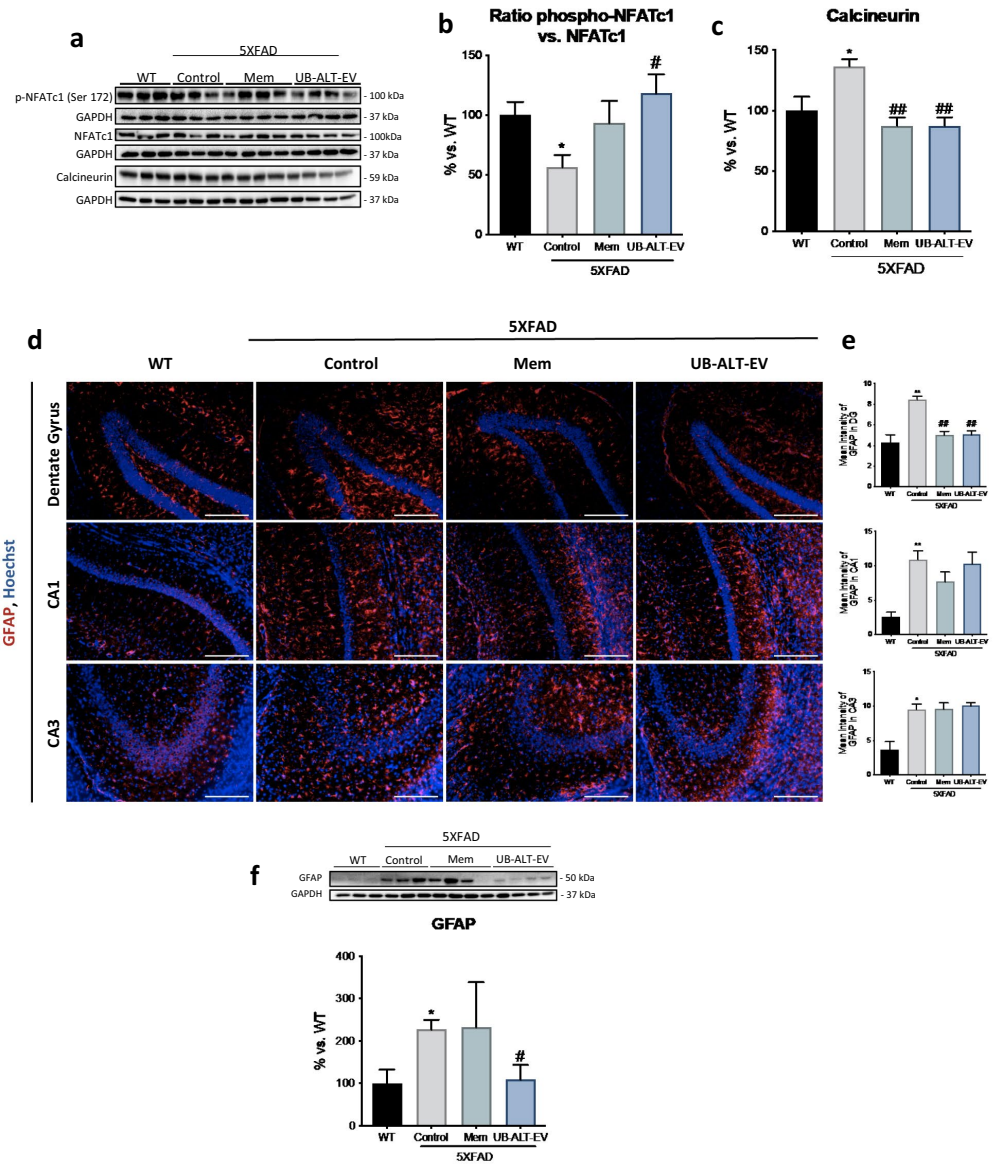
Treatment with UB-ALT-EV changed the protein expression of inflammatory and astrocytic activation markers and in 5XFAD mice. Representative Western Blot (**a**) and quantifications for ratio phospho-NFATc1 vs. NFATc1 (**b**) and CaN (**c**). Values in bar graphs are adjusted to 100% for protein levels of the wild type (WT). Representative GFAP (Red) and Hoechst (Blue) positive staining in dentate gyrus, CA1 and CA3 hippocampal areas (**d**). Scale bars: 200 μm. Mean intensity of GFAP staining in dentate gyrus, CA1 region, and CA3 region (**e**). Representative Western Blot and quantification for GFAP (**f**). Values are the mean ± Standard error of the mean (SEM); (n = 3 for WT and Control groups and n = 4 for Mem and UB-ALT-EV groups. For WT vs. 5XFAD Control groups data was analyzed using a two-tail Student’s t-test, and for 5XFAD groups a standard one-way ANOVA followed by Tukey post-hoc analysis was performed. *p < 0.05; **p < 0.01 for WT vs. Control. ^#^p < 0.05; ^##^p < 0.01 for Mem or UB-ALT-EV vs. Control.

Alterations in CaN/NFAT activity have been associated with glial activation and excitotoxity in 5XFAD^30^. In this regard, it would be interesting to evaluate the ability of NMDAR antagonists to modulate excitotoxicity. Strikingly, we found that the phosphorylated NFATc1 was significantly reduced in 5XFAD mice compared to the WT group. Intriguingly, we found that UB-ALT-EV, but not memantine, increased phosphorylated NFATc1levels, thereby preventing nuclear translocation (Fig. 1a,b). Considering these results, CaN, a calcium-dependent protein with phosphatase activity, revealed higher protein levels in the 5XFAD group compared to the WT group. Conversely, in both treated groups, CaN protein levels were decreased compared to the 5XFAD control group (Fig. 1a,c). Collectively, these results suggest a role of both NMDAR antagonists in modulating CaN/NFAT signaling pathway, by reducing Ca^2+^ entry and its downstream mediators.

### UB-ALT-EV and memantine treatments reduced hippocampal astrogliosis in 5XFAD mice

Next, we assessed the impact of NMDAR antagonists on astrogliosis performing an immunofluorescence assay against glial fibrillary acidic protein (GFAP) (Fig. 1d). The results revealed significantly higher GFAP immunostaining in 5XFAD mice compared to WT mice in the dentate gyrus, CA1 and CA3 regions. UB-ALT-EV and memantine treatments reduced astrogliosis in the dentate gyrus but not in CA1 and CA3 brain regions (Fig. 1d,e). Besides, total GFAP protein expression was evaluated by western blot analysis. As expected, 5XFAD showed significantly higher total protein levels when compared to WT and UB-ALT-EV, but not memantine, treatment reduced significantly GFAP protein levels compared to the 5XFAD control group (Fig. 1f).

### Differential expression levels of microglial markers after UB-ALT-EV or memantine treatment in 5XFAD mice

**Figure 2:**
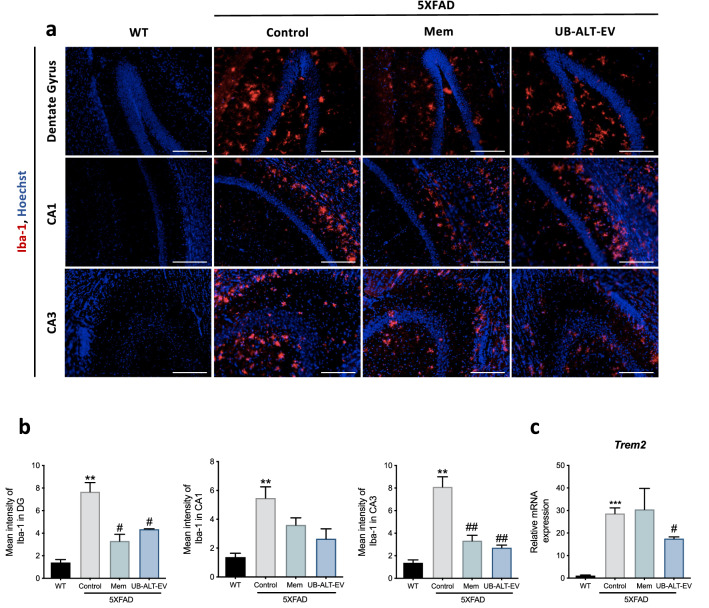
Treatment-induced changes in the expression of microglial activation markers with NMDA receptor antagonists in 5XFAD of 6 months of age. Representative Iba-1 (Red) and Hoechst (Blue) positive staining in dentate gyrus, CA1 and CA3 hippocampal areas (**a**). Scale bars: 200 μm. Mean intensity of Iba-1 staining in dentate gyrus, CA1 region, and CA3 region (**b**). Representative gene expression for *Trem2* (**c**)*.* Values are the mean ± Standard error of the mean (SEM); (n = 3 for WT and Control groups and n = 4 for Mem and UB-ALT-Ev groups. For WT vs. 5XFAD Control groups data was analyzed using a two-tail Student’s t-test, and for 5XFAD groups a standard one-way ANOVA followed by Tukey post-hoc analysis was performed. **p < 0.01; ***p < 0.001 for WT vs. Control. ^#^p < 0.05; ^##^p < 0.01 for Mem or UB-ALT-EV vs. Control.

Signs of microglial activation have been established in the AD brains, indicating a prominent role of neuroinflammation in the pathogenesis of AD^13^. 5XFAD microglial reactivity was evaluated by immunofluorescence analysis of ionized calcium-binding adapter molecule 1 (Iba-1) and *Trem2* gene expression. Higher levels of Iba-1 protein were determined in 5XFAD mice compared to WT mice in CA1, CA3 regions and dentate gyrus (Fig. 2a). NMDAR antagonist treatments reduced Iba-1 immunostaining in dentate gyrus and CA3 regions, but not in CA1 (Fig. 2a,b). Accordingly, *Trem2* gene expression analysis revealed increased expression in 5XFAD mice when compared to WT mice. Interestingly, only UB-ALT-EV treatment was able to significantly reduce gene expression of *Trem2* in 5XFAD mice (Fig. 2c). In this line, the microglial activation marker CD68 was evaluated. Western Blot analysis revealed that NMDA receptor antagonists induced a significant reduction of the protein levels of this microglial marker (Supplementary Fig. 1) in 5XFAD mice. These results demonstrated that UB-ALT-EV reduced microglial markers better than memantine.

### UB-ALT-EV treatment changed the expression of proinflammatory (M1) phenotype microglial markers in 5XFAD mice

**Figure 3:**
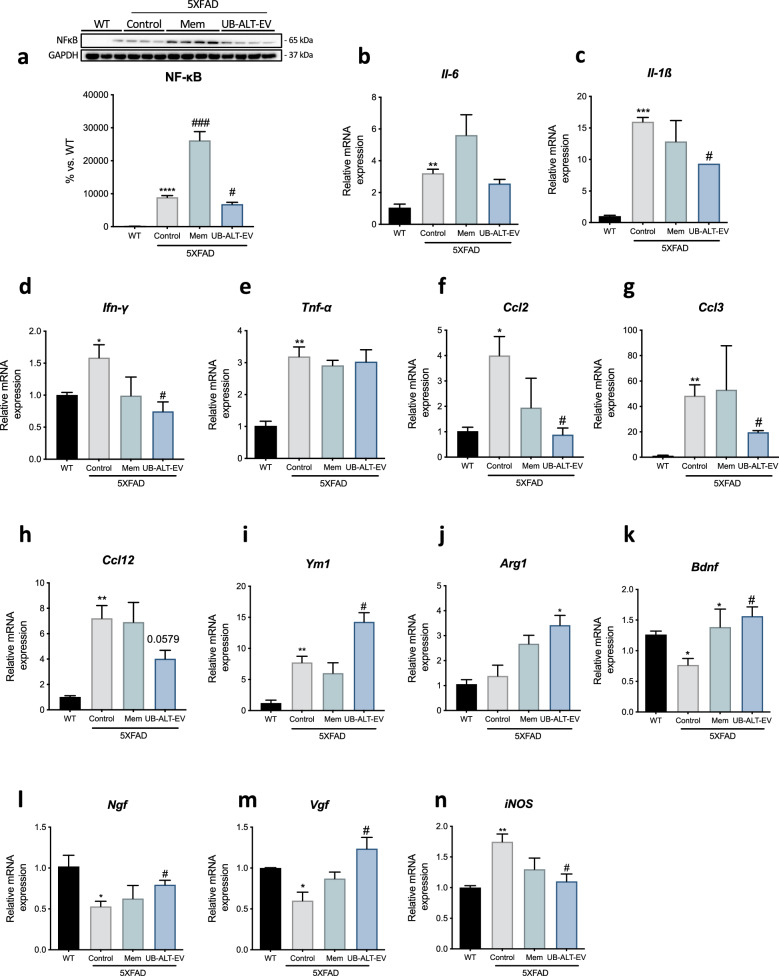
Differential effect of UB-ALT-EV treatment over memantine treatment on gene expression of pro-inflammatory and anti-inflammatory microglial markers in 5XFAD mice. Representative Western Blot and quantification for NF-κB (**a**). Values in bar graphs are adjusted to 100% for protein levels of the wild type (WT). Representative gene expression for *Il-6* (**b**), *Il-1β* (**c**), *Ifn-γ* (**d**), *Tnf-α* (**e**), *Ccl2* (**f**), *Ccl3* (**g**), *Ccl12* (**h**), *Ym1* (**i**), *Arg1* (**j**), *Bdnf* (**k**) *Ngf* (**l**), *Vgf* (**m**) and *iNOS* (**n**)*.* Values are the mean ± Standard error of the mean (SEM); (n = 3 for WT and Control groups and n = 4 for Mem and UB-ALT-EV groups. For WT vs. 5XFAD Control groups data was analyzed using a two-tail Student’s t-test, and for 5XFAD groups a standard one-way ANOVA followed by Tukey post-hoc analysis was performed. *p < 0.05; **p < 0.01; ***p < 0.001; ****p < 0.0001 for WT vs. Control. ^#^p < 0.05; ^###^p < 0.001 for Mem or UB-ALT-EV vs. Control.

Microglial cells can show a damaging "M1 proinflammatory" phenotype or an "M2 anti-inflammatory", neuroprotective phenotype depending on the released mediators. The transcription factor NF-κB promotes the release of inflammatory cytokines/chemokines^31^. Assessment of NF-κB protein levels revealed significantly higher levels in the 5XFAD group compared to WT. Interestingly, while the memantine group significantly increased NF-κB protein levels, UB-ALT-EV treatment in 5XFAD mice significantly reduced protein levels of this transcription factor, compared to the 5XFAD control group (Fig. 3a). To decipher the precise role of NMDARs on inflammation, we carried out an exhaustive gene expression evaluation of proinflammatory markers that are associated with M1-phenotype. Then, we evaluated *Interleukin-6 (Il-6), interleukin-1β (Il-1β)*, *Interferon-gamma (Ifn-γ)*, *tumor necrosis factor α (Tnf-α)*, *monocyte chemoattractant protein-1 (Ccl2)*, *C–C chemokine ligand 3 (Ccl3)* and *C–C Chemokine ligand 12 (Ccl12).* All the proinflammatory markers evaluated revealed significantly higher expression in 5XFAD mice compared to WT mice (Fig. 3b,c,d,e,h). Interestingly, memantine treatment produced no change compared to 5XFAD control group, but the UB-ALT-EV group showed a significant diminution in *Il-1β, Ifn-γ, Ccl2* and *Ccl3* gene expression compared to 5XFAD Control group (Fig. 3c,d,e,f,g)*.* Taken together, these data suggest that UB-ALT-EV treatment reduces microgliosis, unlike memantine treatment, by reducing the NF-κB signaling pathway.

### UB-ALT-EV treatment increased gene expression of microglial markers (M2 phenotype) together with neurotrophins and reduced *iNOS* gene expression in 5XFAD mice

To further assess microglial activation, we measured anti-inflammatory (M2) markers of the microglial phenotype such as *chitinase-like 3 (Ym1)*, *Arginase-1* (*Arg1*)*,* and neurotrophins gene expression. Interestingly, 5XFAD mice showed higher expression levels of *Ym1* compared to WT mice, which may indicate a possible compensatory mechanism (Fig. 3i). Besides, results revealed a significant anti-inflammatory effect after UB-ALT-EV treatment in 5XFAD mice, whereas memantine treatment did not induce changes (Fig. 3i,j). Furthermore, microglial cells could affect neuronal plasticity by releasing neurotrophic factors^32^. Thus, we found that after UB-ALT-EV administration, *Bdnf, Ngf* and *Vgf* gene expression in 5XFAD increased significantly, similar to the levels exhibited by WT mice, whereas for memantine, only *Bdnf* gene expression was increased (Fig. 3k,l,m). These results revealed the robust neuroprotective efficacy of UB-ALT-EV compared to memantine*.* Finally, we investigated the association between neuroinflammaiton and OS through *iNOS* gene expression. Interestingly, we observed that only the UB-ALT-EV treated group could reduce *iNOS* gene expression (Fig. 3n).

### UB-ALT-EV treatment regulates genes associated with OS response in 5XFAD mice

**Figure 4:**
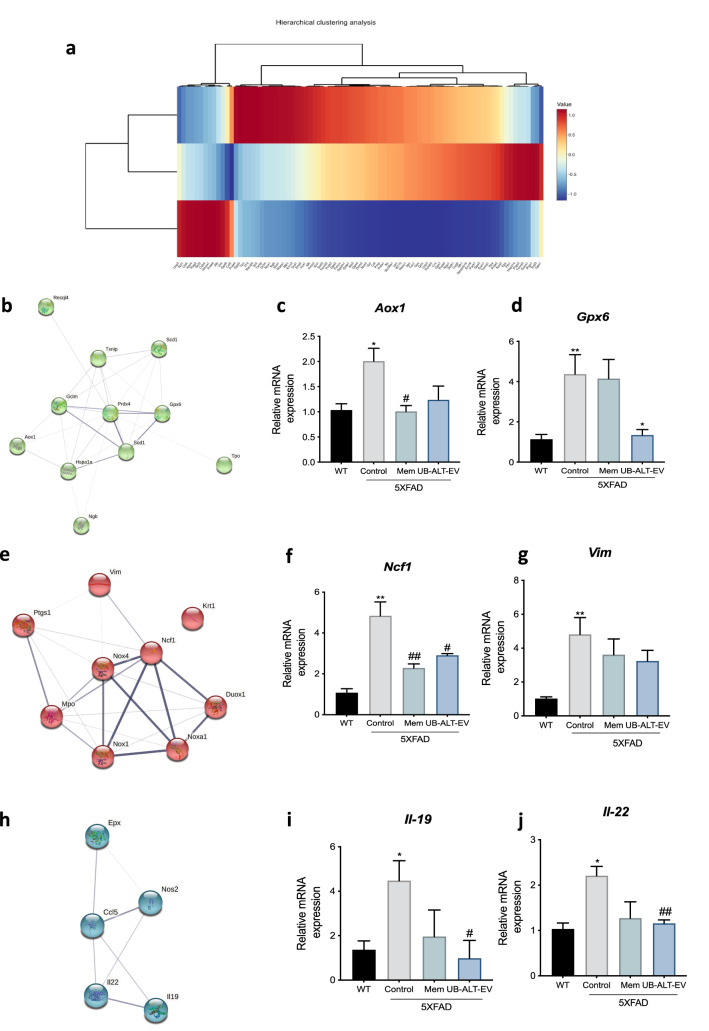
qPCR analysis of 84 OS-related gene expression after a treatment with NMDA receptor antagonists in 6-month-old 5XFAD mice. Hierarchical clustering of the qPCR array in WT and 5XFAD mice treated with UB-ALT-EV (**a**). The predicted protein–protein interactions analysis are shown (**b**, **e**, **h**). Validation of representative subset of genes. Gene expression of *Aox1* (**a**), *Gpx6* (**b**), *Ncf1* (**f**), *Vim* (**g**), *Il-19* (**i**), and *Il-22* (**j**) in the hippocampus of WT and 5XFAD mice groups. Gene expression were determined by qPCR. Values are the mean ± Standard error of the mean (SEM); (n = 3 for WT and Control groups and n = 4 for Mem and UB-ALT-EV groups. For WT vs. 5XFAD Control groups data was analyzed using a two-tail Student’s t-test, and for 5XFAD groups a standard one-way ANOVA followed by Tukey post-hoc analysis was performed. *p < 0.05; **p < 0.01; ***p < 0.001; ****p < 0.0001 for WT vs. Control. ^#^p < 0.05; ^###^p < 0.001 for Mem or UB-ALT-EV vs. Control.

We then sought further to investigate the OS response following treatment with UB-ALT-EV. First, we determined the expression profile of OS-associated genes in the UB-ALT-EV-treated 5XFAD mice, whereby we carried out a qPCR array containing 84 OS-related genes. Clustering analysis revealed three gene clusters (Fig. 4a). After function and process pathway analysis by using the GO database, we found that cluster 1 functional enrichment is mainly associated with superoxide activity (GO:0016175), and oxidoreductase activity (GO:0016491) (Table 1, and Supplementary Fig. 1). Likewise, the process enrichment is associated with superoxide processes and response to OS (Supplementary Fig. 1). The changes in *Aox1* and *Gpx6* gene expression found were also validated for UB-ALT-EV group but nor for memantine (Fig. 4b,c,d). Cluster 2 is functional associated with antioxidant (GO:0016209), oxidoreductase (GO:0016491) and peroxidase (GO:0004601) activities (Table 2, and Supplementary Fig. 1), partially validated through *Ncf1* and *Vim* for both NMDAR antagonist treatments (Fig. 4e,f,g). Besides, the GO enrichment process showed modulation associated with response to OS, stress and ROS metabolic process (Supplementary Fig. 1). Finally, cluster 3 is associated with cytokine activity (GO:0005125) in functional enrichment analysis and mainly associated with ROS metabolic as well as IL-6 production in process enrichment (Table 3, and Supplementary Fig. 1), and it was only validated through *Il-19* and *Il-22* in UB-ALT-EV treated mice group (Fig. 4h,i,j). Additionally, KEGG analysis for cluster 1 demonstrated alterations in the phagosome, and AD pathways, among others. Regarding KEGG analysis for cluster 2, we found enrichment associated with tyrosine and glutathione metabolisms, longevity regulating pathway, among others (Supplementary Fig. 1). By last, KEGG analysis for cluster 3 demonstrated the association with cytokine-cytokine receptor interaction and some inflammatory pathways (Supplementary Fig. 1).

**Table 1:** Function and process pathway analysis by using the GO data base of cluster 1.

#Term ID	Description	Observed gene count	Background gene count	Strength	False discovery rate	Matching proteins in your network (IDs)	Matching proteins in your network (labels)
GO:0016175	Superoxide-generating nad(p)h oxidase activity	3	8	2.96	4.04e-05	10090.ENSMUSP00000016094, 10090.ENSMUSP00000032781, 10090.ENSMUSP00000033610	*Ncf1,Nox4,Nox1*
GO:0016176	Superoxide-generating nadph oxidase activator activity	2	8	2.79	0.0070	10090.ENSMUSP00000016094, 10090.ENSMUSP00000037423	*Ncf1,Noxa1*
GO:0016491	Oxidoreductase activity	5	710	1.24	0.0070	10090.ENSMUSP00000016094, 10090.ENSMUSP00000020779, 10090.ENSMUSP00000032781, 10090.ENSMUSP00000033610,10090.ENSMUSP00000059977	*Ncf1,Mpo,Nox4,Nox1,Ptgs1*
GO:0016174	NAD(P)H oxidase H2O2-forming activity	2	9	2.74	0.0071	10090.ENSMUSP00000032781, 10090.ENSMUSP00000033610	*Nox4,Nox1*
GO:0006801	Superoxide metabolic process	5	33	2.57	2.53e-08	10090.ENSMUSP00000016094, 10090.ENSMUSP00000020779, 10090.ENSMUSP00000032781, 10090.ENSMUSP00000033610,10090.ENSMUSP00000037423	*Ncf1,Mpo,Nox4,Nox1,Noxa1*
GO:0042554	Superoxide anion generation	4	13	2.88	2.66e-07	10090.ENSMUSP00000016094, 10090.ENSMUSP00000032781, 10090.ENSMUSP00000033610, 10090.ENSMUSP00000037423	*Ncf1,Nox4,Nox1,Noxa1*
GO:0006952	Defense response	7	1133	1.18	0.00014	10090.ENSMUSP00000016094, 10090.ENSMUSP00000020779, 10090.ENSMUSP00000023790, 10090.ENSMUSP00000028062, 10090.ENSMUSP00000032781, 10090.ENSMUSP00000033610, 10090.ENSMUSP00000059977	*Ncf1,Mpo,Krt1,Vim,Nox4,Nox1,Ptgs1*
GO:0042743	Hydrogen peroxide metabolic process	3	33	2.35	0.0012	10090.ENSMUSP00000016094, 10090.ENSMUSP00000020779, 10090.ENSMUSP00000033610	*Ncf1,Mpo,Nox1*
GO:0002679	Respiratory burst involved in defense response	2	5	2.99	0.0091	10090.ENSMUSP00000016094, 10090.ENSMUSP00000020779	*Ncf1,Mpo*
GO:0006979	Response to oxidative stress	4	395	1.39	0.0309	10090.ENSMUSP00000016094, 10090.ENSMUSP00000020779, 10090.ENSMUSP00000033610, 10090.ENSMUSP00000059977	*Ncf1,Mpo,Nox1,Ptgs1*
GO:0001878	Response to yeast	2	14	2.54	0.0310	10090.ENSMUSP00000016094, 10090.ENSMUSP00000020779	*Ncf1,Mpo*
GO:1903409	Reactive oxygen species biosynthetic process	2	19	2.41	0.0452	10090.ENSMUSP00000016094, 10090.ENSMUSP00000020779	*Ncf1,Mpo*

**Table 2:** Function and process pathway analysis by using the GO data base of cluster 2.

#Term ID	Term description	Observed gene count	Background gene count	Strength	False discovery rate	Matching proteins in your network (IDs)	Matching proteins in your network (labels)
GO:0016209	Antioxidant activity	4	70	2.06	0.00020	10090.ENSMUSP00000004453, 10090.ENSMUSP00000021005, 10090.ENSMUSP00000023707, 10090.ENSMUSP00000026328	*Gpx6,Tpo,Sod1,Prdx4*
GO:0016491	Oxidoreductase activity	6	710	1.23	0.0012	10090.ENSMUSP00000001027, 10090.ENSMUSP00000004453, 10090.ENSMUSP00000021005, 10090.ENSMUSP00000023707, 10090.ENSMUSP00000026328, 10090.ENSMUSP00000036936	*Aox1,Gpx6,Tpo,Sod1,Prdx4,Scd1*
GO:0004601	Peroxidase activity	3	40	2.18	0.0020	10090.ENSMUSP00000004453, 10090.ENSMUSP00000021005, 10090.ENSMUSP00000026328	*Gpx6,Tpo,Prdx4*
GO:0006979	Response to oxidative stress	6	395	1.48	0.00026	10090.ENSMUSP00000004453, 10090.ENSMUSP00000021005, 10090.ENSMUSP00000023707, 10090.ENSMUSP00000026328, 10090.ENSMUSP00000029769, 10090.ENSMUSP00000102710	*Gpx6,Tpo,Sod1,Prdx4,Gclm,Txnip*
GO:0006950	Response to stress	9	3045	0.77	0.0023	10090.ENSMUSP00000004453, 10090.ENSMUSP00000021005, 10090.ENSMUSP00000023707, 10090.ENSMUSP00000026328, 10090.ENSMUSP00000029769, 10090.ENSMUSP00000036936, 10090.ENSMUSP00000044363, 10090.ENSMUSP00000084586, 10090.ENSMUSP00000102710	*Gpx6,Tpo,Sod1,Prdx4,Gclm,Scd1,Recql4,Hspa1a,Txnip*
GO:0072593	Reactive oxygen species metabolic process	3	101	1.77	0.0318	10090.ENSMUSP00000021005, 10090.ENSMUSP00000023707, 10090.ENSMUSP00000026328	*Tpo,Sod1,Prdx4*

**Table 3:** Function and process pathway analysis by using the GO data base of cluster 3.

#Term ID	Term description	Observed gene count	Background gene count	Strength	False discovery rate	Matching proteins in your network (IDs)	Matching proteins in your network (labels)	
GO:0005125	Cytokine activity	3	215	1.79	0.0491	10090.ENSMUSP00000039600, 10090.ENSMUSP00000094449, 10090.ENSMUSP00000108084	*Ccl5,Il22,Il19*	
GO:0072593	Reactive oxygen species metabolic process	4	101	2.24	4.23e-05	10090.ENSMUSP00000018610, 10090.ENSMUSP00000050497, 10090.ENSMUSP00000094449, 10090.ENSMUSP00000108084	*Nos2,Epx,Il22,Il19*	
GO:0032635	Interleukin-6 production	2	12	2.87	0.0327	10090.ENSMUSP00000018610, 10090.ENSMUSP00000108084	*Nos2,Il19*	
GO:0072677	Eosinophil migration	2	18	2.69	0.0455	10090.ENSMUSP00000039600, 10090.ENSMUSP00000050497	*Ccl5,Epx*	

On the other hand, using the TRRUST database, we found that some genes present in cluster 1, 2 and 3 can be regulated by NF-kB and REL (Supplementary Table 2), two subunits of the transcription factor NF-kB, suggesting that NF-kB modulates the neuroinflammatory process in the 5XFAD model after UB-ALT-EV treatment. Therefore, these results suggest that treatment with UB-ALT-EV in 5XFAD reverses the OS process, affecting the neuroinflammation and vice versa in a better way than memantine treatment.

## Discussion

Based on our previous studies demonstrating the promising role of UB-ALT-EV, an NMDA antagonist, against neurodegeneration^28^, we hypothesized that this new compound could have an additional therapeutic role in neuroinflammation. As a relevant key mechanism in the onset of AD, apart from the canonical calcium signaling regulation mediated by NMDA antagonists. We found that UB-ALT-EV promoted neuroprotection by modifying neuroinflammation, reducing glial activation via microglial response modulation. Moreover, UB-ALT-EV exhibited a relevant role in the reduction of the OS state, due to the up-regulation of anti-inflammatory mediators in an AD mouse model (5XFAD).

Brain inflammation is a defense mechanism with a neuroprotective effect against injury and infection, and in early degenerative stages, there is a balance between proinflammatory and anti-inflammatory responses^33^. However, there appears to be a shift from resting microglia to classically activated microglia in AD, impairing the cytokine balance that ultimately leads to a chronic neuroinflammatory environment^34^. Otherwise, there are several pathways by which Ca^2+^ dysregulation can be linked to releasing cytokines that promote neuroinflammation. In particular, the CaN/NFAT pathway has been associated with Ca^2+^ dysregulation and glial activation^35^. The increase in the dephosphorylated form of the transcription factor NFATc1 reflects an increase in its nuclear translocation and fosters a chronic inflammatory state, synapse dysfunction and glutamate dysregulation^36^. CaN, as a phosphatase, can dephosphorylate NFAT allowing its nuclear translocation and the activation of cytokines transcription. These observations are consistent with several published reports showing high CaN and NFAT protein levels in 5XFAD^30^. However, to our knowledge, the effect of NMDAR blockage on CaN/NFAT pathway remains unknown. In our study, we found that both NMDAR antagonists tested, memantine and UB-ALT-EV reduced CaN protein levels but only UB-ALT-EV significantly increased p-NFATc1/NFAT protein level ratio. Therefore, these results suggest the implication of UB-ALT-EV in preventing the production of several proinflammatory cytokines regulated by CaN/NFAT signaling pathway, and likewise a diminution in gliosis phenomena.

In the light of our findings, we further explored astrocyte activation by evaluating GFAP protein levels in the hippocampus. As expected, an up-regulation of GFAP protein was observed in the hippocampus of 5XFAD mice^37–39^. Memantine significantly reduced GFAP protein levels in the dentate gyrus, while UB-ALT-EV delivered similar results. In addition, UB-ALT-EV treatment significantly reduced total GFAP protein content, probably because the reduction in global astroglial activation was greater after UB-ALT-EV treatment than with memantine.

Microglial activation is known to be divided into two phenotypic profiles: the proinflammatory (M1) and the anti-inflammatory (M2)^40^. In AD, there is an altered homeostasis between the two microglial phenotypes, and a cytokine-dominated inflammatory landscape occurs, enhancing the shift to the M1 phenotype over M2^41^. Therefore, to pursue the inflammatory process accounting in 5XFAD mice model, the microglial status was evaluated through Iba-1, *Trem2* and CD68. According to the literature, increases in Iba-1 were found in 5XFAD mice^37^. Strikingly, we observed a reduction in Iba-1 staining in 5XFAD hippocampus treated with NMDAR antagonists, specifically in the CA3 region^26,42^. TREM2, a cell surface receptor that is mainly expressed in microglial cells^43^, is relevant in the phagocytic role of microglia, constituting a key mediator against Aβ deposition^44^. As a consequence of glial reactivity in 5XFAD mice, an up-regulation of the *Trem2* gene expression accounted^37^. Interestingly, we found that UB-ALT-EV but not memantine induced changes in *Trem2* gene expression, suggesting a novel anti-inflammatory effect of UB-ALT-EV by preventing microglial activation. In line with our results, a recent study in microglial cells demonstrated that TREM2 levels were unchanged after memantine pretreatment^45^. This phagocytosis-reducing effect was also demonstrated by the evaluation of the CD68 marker^46^.

NF-κB is also a CaN-sensitive transcription factor implicated in the proinflammatory phenotype (M1) of glial activation^47^. Recent studies reported the NF-κB inhibition by memantine indicating that NF-κB signaling can be regulated after NMDARs^48,49^. Here, we found an unexpected up-regulation of NF-κB in the 5XFAD memantine-treated group, being downregulated in the case of UB-ALT-EV treatment, accordingly with arrays results by the TRRUST analysis. Thus, these results indicated a different modulation of the inflammatory process by the two NMDARs antagonist tested.

To delve further into these findings, several proinflammatory cytokines associated not only with M1 microglial phenotype, but also with astrocytic activation, were evaluated. Interestingly, *Ifn-γ*, *Il-1β*, *Ccl2* and *Ccl3* gene expression was reduced only by UB-ALT-EV in the 5XFAD group correlating with reduced NF-κB protein levels. Of note, the anti-inflammatory activated microglia (M2) phenotype can contribute to neuronal plasticity by also expressing neurotrophic factors^40,41,47^. Here, when M2 microglial state was assessed in 5XFAD mice, UB-ALT-EV treatment induced not only up-regulation of *Ym1* and *Arg1* gene expression but also the up-regulation of several neurotrophins such as *Bdnf, Ngf* and *Vgf,* involved in neuroprotection^50^, whereas memantine only induced the expression of *Bdnf*, indicating the improved capacity of UB-ALT-EV to promote neuroprotective effects. Consistent with these findings, we previously demonstrated improvements in the reduction of Aβ deposition, tau hyperphosphorylation, and autophagic processes after UB-ALT-EV treatment compared to the memantine group^28^.

Lastly, we found that *iNOS* gene expression was modulated in a significant way by UB-ALT-EV, and weakly by memantine. iNOS is an enzyme implicated both in the control of inflammatory mediators’ release, but also in the generation of ROS that contributes to neuronal damage and AD progression^17^. Besides, it is well-established that neuroinflammation can induce OS, and vice versa, by activating multiple pathways. Then, we evaluated the implication of OS modulation in the neuroprotective effects of UB-ALT-EV by using a qPCR array determination of the 84 genes associated with OS signaling pathways. After analysis, we found that UB-ALT-EV changed the gene expression of 24 genes in 5XFAD, generating three hierarchical clusters based on the enrichment heatmap. Remarkably, our results showed alterations in GO enrichment analysis in processes associated with superoxide-generating NADPH oxidase activity (Cluster 1), cytokine activity (Cluster 2), and antioxidant activity (Cluster 3), among others. KEGG pathways are also related to OS and inflammation, among others. Furthermore, we were able to validate that the gene expression of *Il-19*, *Il-22*, *Gpx6*, and *Ncf1* were significantly reduced in 5XFAD treated mice, and a clear tendency was observed for *Aox1* and *Vim.* Gene array validation indicated a better modulation of UB-ALT-EV than memantine, for both OS and inflammatory pathways, thus demonstrating optimization of the efficacy of the NMDAR antagonist.

**Figure 5:**
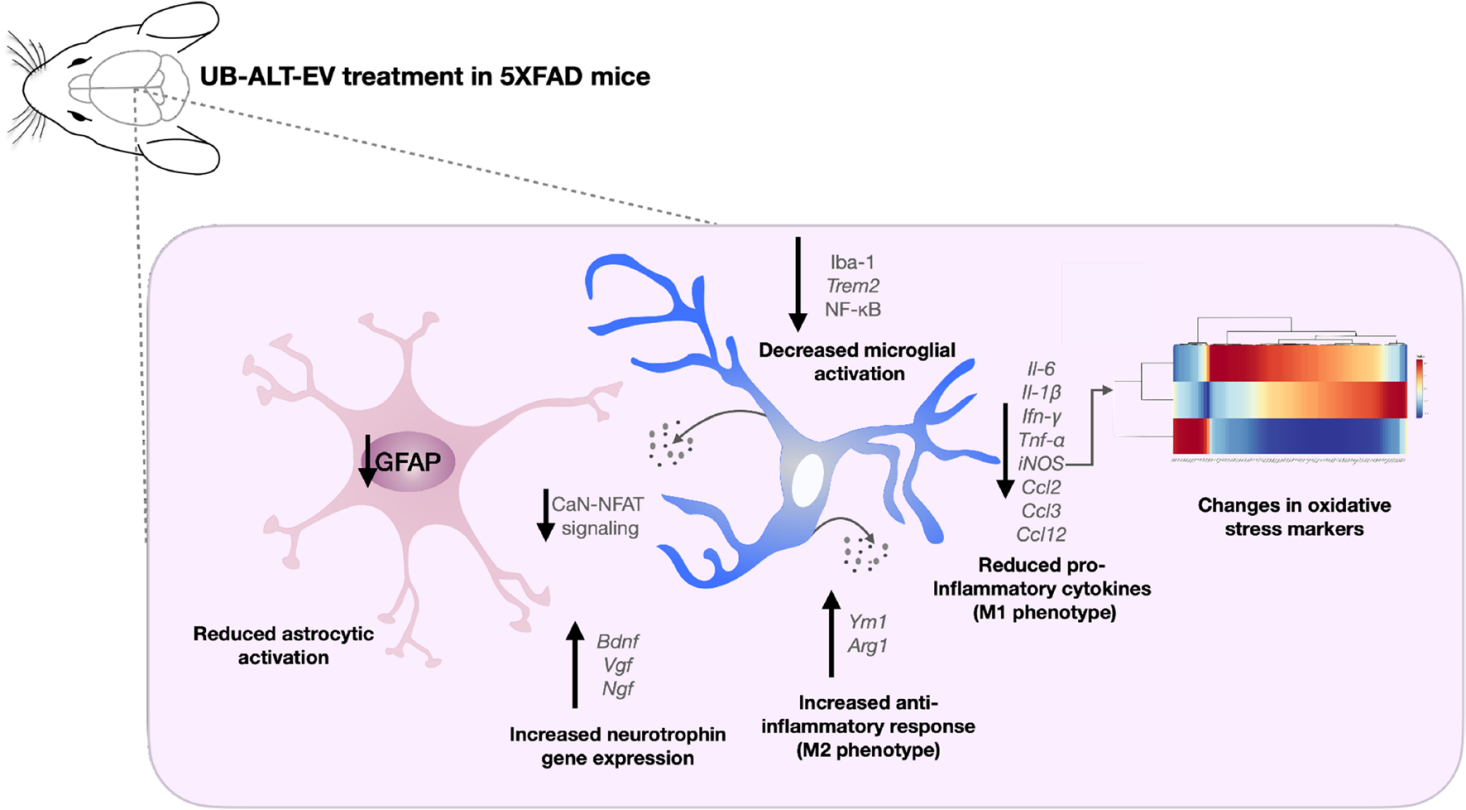
Illustrative cartoon of effects of UB-ALT-EV in 6-month-old 5XFAD mice in oxidative and neuroinflammatory pathology. This figure was generated with Keynote for macOS (ver. 12.1).

The use of AD transgenic animals is a useful tool to evaluate AD pathology, but it must be kept in mind that they do not represent the disease in all its aspects and are by themselves a limitation in the research. This limitation has to be present also in the present work in which one of the most widely used animal model in AD research was used: the 5XFAD. 5XFAD develop amyloid pathology along with cognitive impairment but the NFTs formation is not characteristic of this model^51,52^. Overall, our results highlighted that the treatment with UB-ALT-EV changes the inflammatory landscape in 5XFAD mice. Besides, anti-inflammatory effects were accompanied by OS changes. These beneficial effects were not so clear in 5XFAD treated with memantine, indicating additional pharmacological activities of UB-ALT-EV, which were also found in previous studies^28^. Thus, the optimized NMDARs antagonist, UB-ALT-EV, offers a new opportunity for the treatment of AD, showing novel neuroprotective mechanisms including the reduction of proinflammatory microglial markers and the amelioration of astrocyte activation, additionally to calcium entry blockage (Fig. 5).

## Methods

### Animals and treatment

Six-month-old female 5XFAD mice (n = 29) and wild type (WT) (n = 14) mice were randomly allocated to experimental groups and divided into four groups: WT control and 5XFAD control, administrated with vehicle ((2-hydroxypropyl)-β-cyclodextrin 1.8%) and 5XFAD treated with memantine, or UB-ALT-EV (5 mg/kg/day) dissolved in vehicle. The sample size for the intervention was chosen following previous studies in our laboratory and using one of the available interactive tools (http://www.biomath.info/power/index.html) that is designed to estimate the required sample size to achieve adequate power. Compounds were administered through drinking water for 4 weeks and the doses of each cage were weekly re-calculated taking into account the daily water consumption in each cage and monitoring the body weight of the animals. Animals had free access to food and water and were maintained under standard temperature conditions (22 ± 2 °C) and 12 h: 12 h light–dark cycles (300 lx/0 lx). Water consumption was monitored weekly, and both NMDAR antagonist concentrations were adjusted in the tap water bottle to achieve the precise dose^53^.

Research was conducted in accordance with the Institutional Guidelines for the Care and Use of Laboratory Animals established by (European Communities Council Directive 2010/63/EU and Guidelines for the Care and Use of Mammals in Neuroscience and Behavioural Research, National Research Council 2003) and approved by the Ethics Committee for Animal Experimentation (CEEA) of the University of Barcelona. All experimental procedures with mice followed the recommendations in the ARRIVE guidelines^54^. All possible efforts were taken to reduce the number of animals and their suffering.

### RNA extraction and gene expression determination

Mice were euthanized by cervical dislocation after the treatment period. The brains were immediately removed from the skulls, and the hippocampus were dissected, frozen and maintained at − 80 °C. Total RNA isolation from hippocampal samples was performed using the TRIzol reagent according to the manufacturer’s instructions (Bioline Reagent). The yield, purity and quality of RNA were determined spectrophotometrically with a NanoDrop ND-1000 apparatus (Thermo Scientific) and an Agilent 2100B Bioanalyzer (Agilent Technologies). RNA samples with 260/280 ratios higher than 1.9 were selected. Reverse Transcription-Polymerase Chain Reaction (RT-PCR) was performed. Briefly, 1 μg and 2 μg of messenger RNA (mRNA) was reverse transcribed using a high-capacity cDNA reverse transcription kit (Applied Biosystems) for PCR Array performance and q-PCR validation, respectively. Genes are listed in Supplementary Table 1.

SYBR Green real-time PCR was performed on a Step One Plus Detection System (Applied-Biosystems) employing SYBR Green PCR Master Mix (Applied-Biosystems). Each reaction mixture contained 6.75 μL of complementary DNA (cDNA) (which concentration was 2 μg), 0.75 μL of each primer (which concentration was 100 nM), and 6.75 μL of SYBR Green PCR Master Mix (2X). Data were analyzed utilizing the comparative Cycle threshold (Ct) method (ΔΔCt), where the housekeeping gene level was used to normalize differences in sample loading and preparation. Normalization of expression levels was performed with *β-actin* for SYBR Green-based real-time PCR results. Each sample was analyzed in duplicate, and the results represent the n-fold difference of the transcript levels among different groups.

### Real-time quantitative PCR array

Real-time quantitative PCR array containing 84 oxidative stress-related genes (qPCR Sign Arrays 96 system, AnyGenes, Paris, France) was used for screening according to the instructions of the manufacturer. Briefly, 2 μL of diluted cDNA pooled samples (n = 3–4) (2 μg cDNA diluted at the 1/12 from Reverse Transcription (20 μL) performed with 1 μg of RNA) was mixed with 10 μl of 2 × Perfect Master Mix SYBR Green and 8 μL Ultra-pure H_2_O and added to each well, being consequently the total reaction volume 20 μL per well. After 20 μL of the reaction mix was in each well of the 96-well plate, the plate was centrifuged and then the qPCR run was performed using a Step One Plus Detection System (Applied-Biosystems), following the manufacturer’s recommendations and protocols. PCR reaction conditions were 95 °C, 10 min; 95 °C, 5 s and 60 °C, 30 s, × 40 cycles. After completion of the reaction, the melting curve was analyzed, 95 °C, 10 s, 65 °C–95 °C, 30 s.

### Hierarchical clustering

Hierarchical clustering was carried out with the genes screened on the qPCR Sign Arrays 96 system to assess the expression profile among the study groups. Genes were grouped into three clusters based on the expression profile using the R package pheatmap. Expression data were then clustered by Euclidean distances between genes and by applying the complete hierarchical clustering method.

### Protein–protein interaction network and functional annotation

We conducted protein–protein interaction networks using the STRING database^55^. A PPI enrichment p-value < 0.001 was taken as statistically significant, indicating that proteins are at least partially biologically connected. To establish the functional annotation of the three groups, we identified the Gene Ontology (GO) and undertook pathway analysis with the Kyoto Encyclopedia of Genes and Genomes (KEGG), by using the Database for Annotation, Visualisation and Integrated Discovery (DAVID)^56^. GO terms and KEGG pathways with an adjusted p-value < 0.05 were taken as statistically significant. We used the KEGG mapping tool to show downregulated (green) and up-regulated (red) genes in the KEGG pathway maps^57^. To assess transcriptional regulatory interactions between the three gene clusters and mouse transcriptional factors (TFs), we used the TRRUST database^58^. TRRUST identifies potential TFs involved in the regulation of genes of interest. TFs with an adjusted p-value < 0.05 were considered statistically significant.

### Protein level determination by Western blotting

For western blot analyses, mice were euthanized by cervical dislocation and the brains were removed immediately from the skull. The hippocampus was then excised and frozen in powdered dry ice. They were kept at − 80 °C for subsequent use in protein extraction. For protein extraction, tissue samples were homogenized in lysis buffer containing phosphatase and protease inhibitors (Cocktail II, Sigma-Aldrich). Total protein levels were extracted, protein concentration was determined by Bradford's method and 15 μg samples were separated by sodium dodecyl sulphate–polyacrylamide dodecyl sulphate gel electrophoresis (SDS-PAGE) (8–20%). After transfer to polyvinylidene difluoride (PVDF) membranes (Millipore), the membranes underwent blocking in 5% nonfat milk in Tris-buffered saline (TBS) containing 0.1% Tween 20 TBS (TBS-T) for 1 h at room temperature (RT), which was followed by overnight incubation at 4 °C with the primary antibodies: NFATc1 (St John’s/STJ24751) diluted 1:1000; p-NFAT (Ser172) (Invitrogen/PA5-64696) diluted 1:500; Calcineurin (BioRad/VPA00329) diluted 1:1000; Glyceraldehyde-3-Phosphate Dehydrogenase (Millipore/MAB374) diluted 1:5000; GFAP (Gene Tex/GTX100850) diluted 1:1000; NF-κB (Cell signaling/8242S) diluted 1:1000; Goat-anti-mouse HRP conjugated (Biorad Lab 170-5047) diluted 1:6000 and Goat-anti-rabbit HRP conjugated (Biorad Lab/170-6515) diluted 1:6000). The membranes were then washed and incubated with secondary antibodies for 1 h at RT. Immunoreactive proteins were imaged with the chemiluminescence-based detection kit, following the manufacturer's protocol (ECL Kit, Millipore), and digital images were then acquired using the ChemiDoc XRS + system (BioRad). Semi-quantitative analyses were conducted using ImageLab software (BioRad), and results were extracted in arbitrary units (AU), considering control protein levels as 100%. Protein loading was routinely controlled by immunodetection of glyceraldehyde-3-phosphate dehydrogenase (GAPDH).

### Immunofluorescence assay

For immunofluorescence, mice were anesthetized (ketamine 100 mg/kg and xylazine 10 mg/kg, intraperitoneally) and then intracardially perfused with 4% paraformaldehyde (PFA) diluted in 0.1 M phosphate buffer solution. The brains were removed and post-fixed in 4% PFA overnight at 4 °C. Thereafter, the brains were changed to PFA + 15% sucrose. Finally, brains were frozen in powdered dry ice and stored at − 80 °C until sectioning. Coronal brain sections of 30 μm were obtained (Leica Microsystems CM 3050S cryostat, Wetzlar, Germany) and stored in cryoprotective solution at − 20 °C until used.

Free-floating brain slices were washed 5 min in PBS and blocked and permeabilized in PBS, BSA 1% and 0,3% Triton X-100 solution for 20 min. After two washes of 5 min with PBS (0.1 M), primary antibody (GFAP (Dako/Z0334) or Iba-1 (Abcam/ab48050), were incubated over-night at 4ºC at a dilution of 1:400. The following day, after two washes with PBS, the secondary antibody (Alexa Fluor 594, Abcam/ab150080) was incubated at room temperature for 1 h in the dark at a dilution of 1:400. Later, sections were co-incubated with 1 mg/mL Hoechst (Sigma) staining solution for 5 min in the dark at room temperature and washed twice for 5 min in PBS. Finally, the slices were mounted with Fluoromount G (EMS, USA).

### Image acquisition and analysis

Acquisition of images was conducted with a fluorescence laser microscope (Olympus BX51, Germany). At least 3 sections from 3 different individuals per group were analyzed with ImageJ/Fiji software from the National Institutes of Health available online. We selected similar and comparable histological regions, focusing on the adjacent position of the entire cortical area and the hippocampus of one cerebral hemisphere. For the acquisition of glial fibrillary acidic protein (GFAP) and Iba-1 images, a fixed exposure was maintained for all samples in all the experiments. Fluorescence intensity of GFAP and Iba-1 positive cells was measured in different areas of the hippocampus (CA1, CA3 and dentate gyrus) and the quantification was then averaged from each subject's three different sections.

### Data acquisition and statistical analysis

Data analysis was conducted using GraphPad Prism ver. 9 statistical software. Data are expressed as the mean ± standard error of the mean (SEM) of at least 3 samples per group. Strain was compared using the two tail Student’s t-test to evaluate differences between WT *vs*. 5XFAD control group. For 5XFAD mice One-way analysis of variance (ANOVA) was followed by Tukey post-hoc analysis or two-tail Student’s t-test when necessary. Statistical significance was considered when p-values were < 0.05. The statistical outliers were determined with Grubbs’ test and, when necessary, were removed from the analysis.

## Supplementary Information

Supplementary Figures.

Supplementary Information.

Supplementary Table 1.

Supplementary Table 2.

## Data Availability

The datasets generated and/or analysed during the current study are available in the https://www.ncbi.nlm.nih.gov/geo/ repository, GSE20206. Correspondence and requests for materials should be addressed to C.G.-F.
