# Supplementary material for: Glial cell reactivity and oxidative stress prevention in Alzheimer’s disease mice model by an optimized NMDA receptor antagonist
Source: Sci Rep. 2022 Oct 25;12:17908. doi: 10.1038/s41598-022-22963-x (PMC9596444; doi:10.1038/s41598-022-22963-x)

Supplementary Fig. 1. Representative Western Blot and quantification for CD68 **(a)**. GO database results: Cluster 1 (**b,c**), Cluster 2 (**d,e**), Cluster 3 (**f,g**). KEGG analysis for Cluster 1 (**h**), Cluster 2 (**i**) and Cluster 3 (**j**).

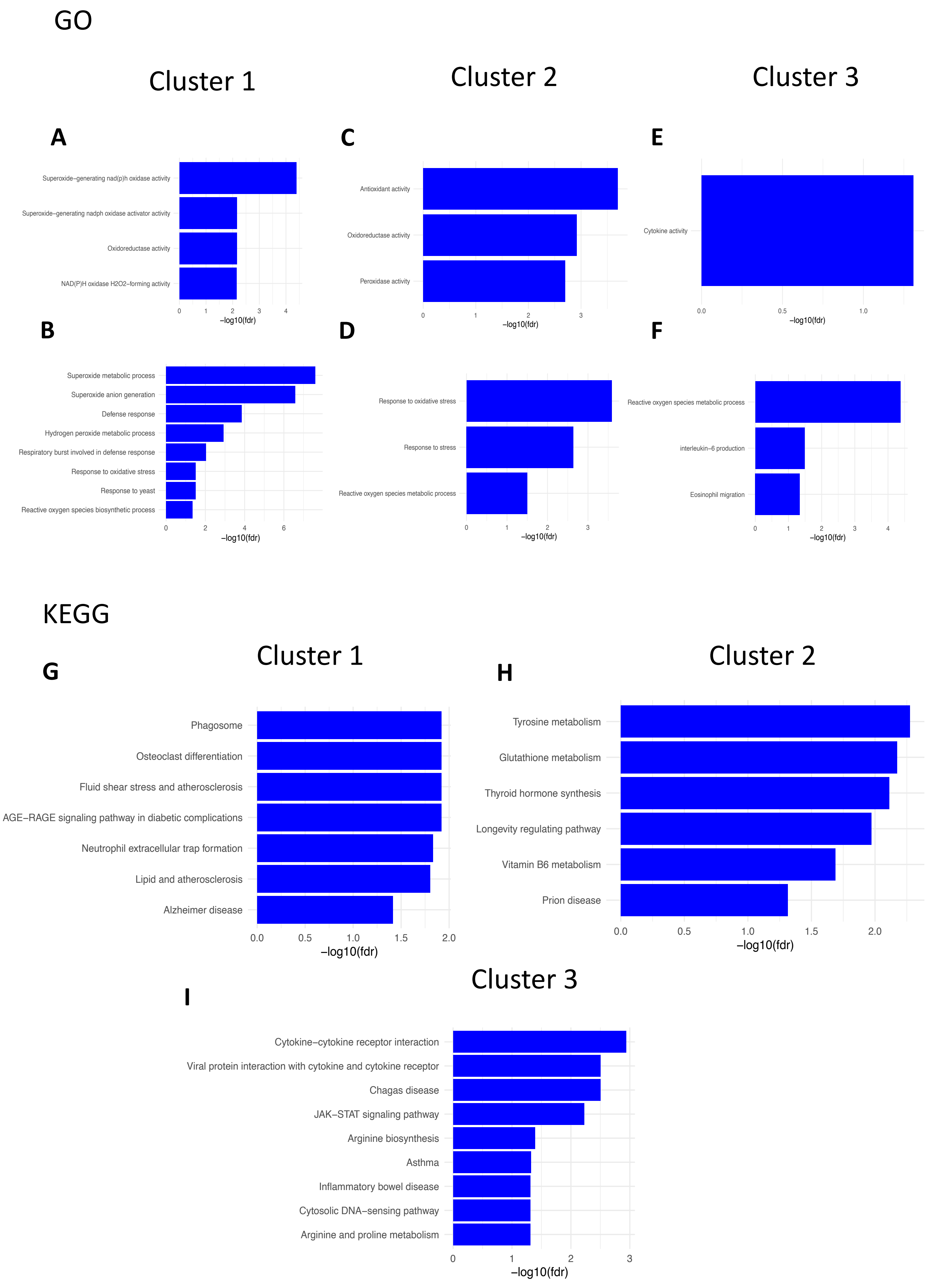

Supplement: Supplementary file 1 — Supplementary Figures. [file 41598_2022_22963_MOESM1_ESM.pdf]
