# Supplementary material for: Glial cell reactivity and oxidative stress prevention in Alzheimer’s disease mice model by an optimized NMDA receptor antagonist
Source: Sci Rep. 2022 Oct 25;12:17908. doi: 10.1038/s41598-022-22963-x (PMC9596444; doi:10.1038/s41598-022-22963-x)

Calcineurin

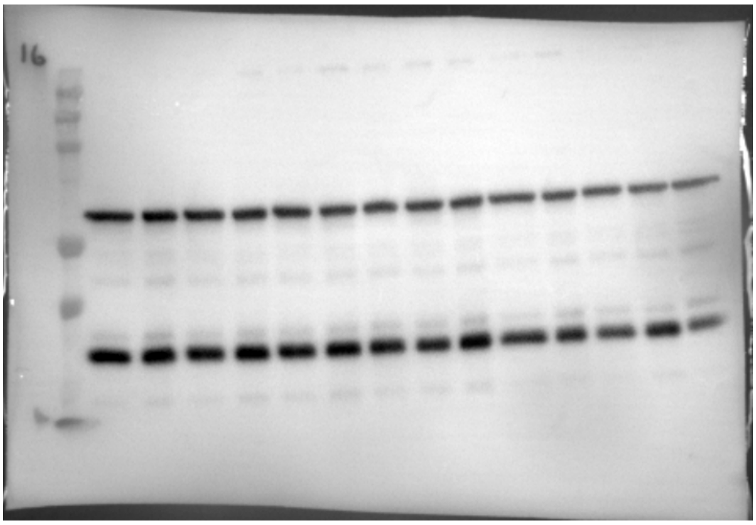

GAPDH for Calcineurin

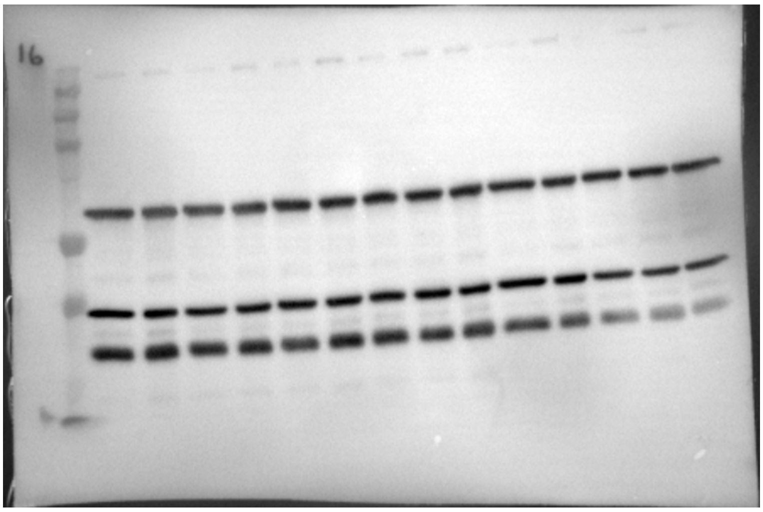

NFAT

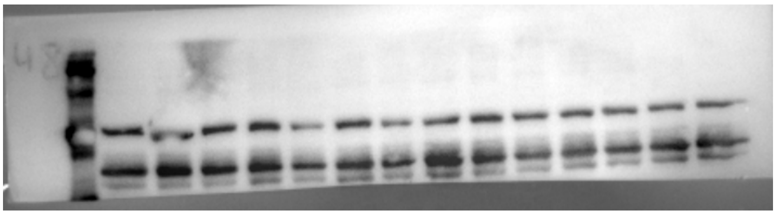

GAPDH for NFAT

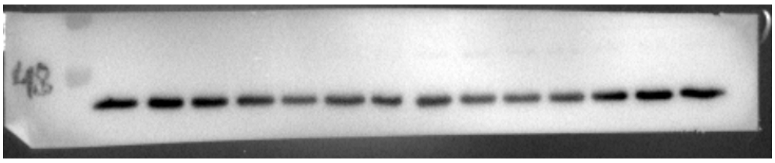

p-NFAT

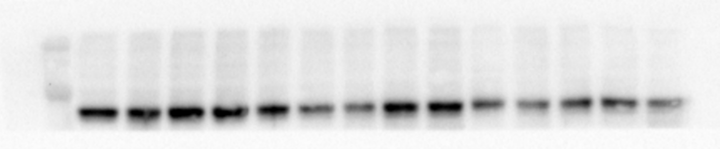

GAPDH for p-NFAT

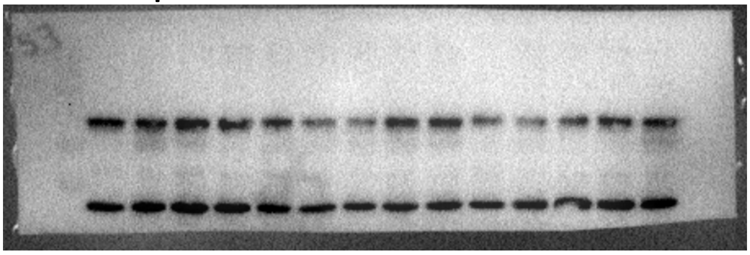

GFAP

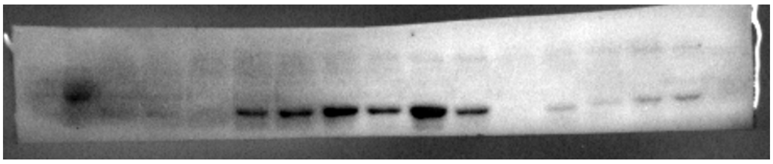

GAPDH for GFAP

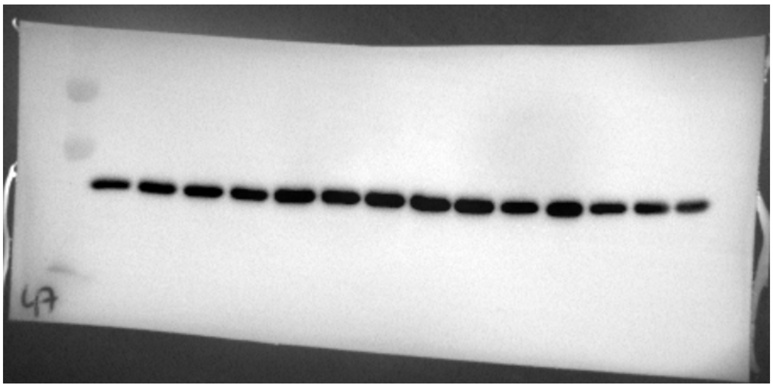

NF-KB

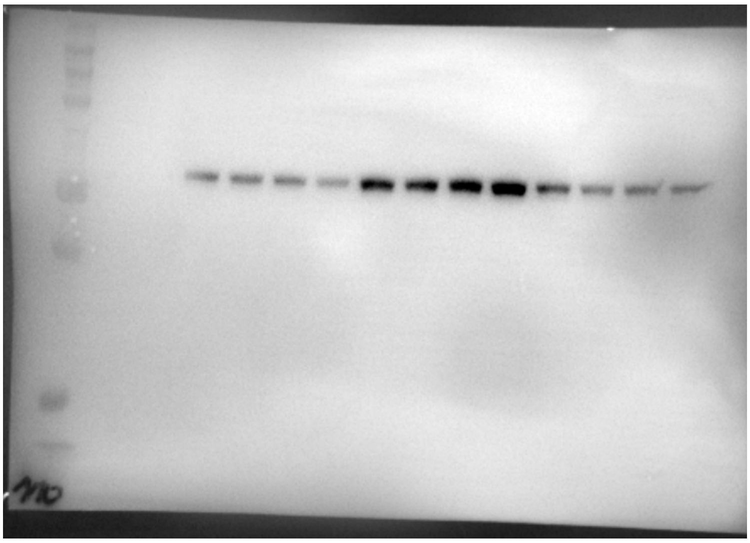

GAPDH for NF-KB

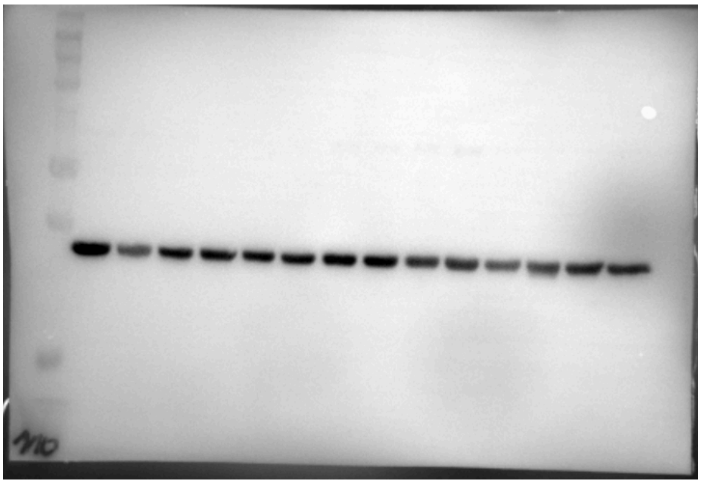

CD68

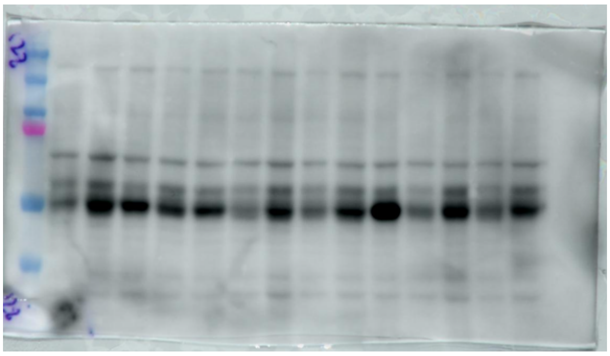

GAPDH for CD68

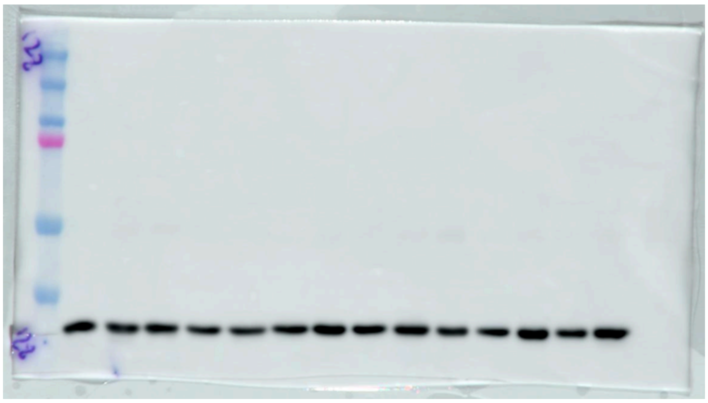

Supplement: Supplementary file 2 — Supplementary Information. [file 41598_2022_22963_MOESM2_ESM.pdf]
