# Supplementary material for: Glial cell reactivity and oxidative stress prevention in Alzheimer’s disease mice model by an optimized NMDA receptor antagonist
Source: Sci Rep. 2022 Oct 25;12:17908. doi: 10.1038/s41598-022-22963-x (PMC9596444; doi:10.1038/s41598-022-22963-x)
Supplement: Supplementary file 3 — Supplementary Table 1. [file 41598_2022_22963_MOESM3_ESM.docx]

**Supplementary Table 1**. Primers used in qPCR studies.

| Target | Forward primer (5'-3') | Reverse primer 5'-3' |
| --- | --- | --- |
| *Trem2* | CCTGAAGAAGCGGAATGGG | CTTGATTCCTGGAGGTGCT |
| *Il-6* | ATCCAGTTGCCTTCTTGGGACTGA | TAAGCCTCCGACTTGTGAAGTGGT |
| *Il-1β* | ACAGAATATCAACCAACAAGTGATATTCTC | GATTCTTTCCTTTGAGGCCCA |
| *Ifn-γ* | CCTTCTTCAGCAACAGCAAGGCG | CTTGGCGCTGGACCTGTGGG |
| *Tnf-α* | TCGGGGTGATCGGTCCCCAA | TGGTTTGCTACGACGTGGGCT |
| *Ccl2* | CCAGCAAGATGATCCAATG | CTTCTTGGGGTCAGCACAGA |
| *Ccl3* | TGACCCCCAAGGCTCAAATA | CCCAGGTCCTCGCTTATGATC |
| *Ccl12* | ACACTGGTTCCTGACTCCTCT | ACCTGAGGACTGATGGTGGT |
| *Ym1* | GACAGGGCTCCTTTCAGGAC | GCCAAGGTTAAAGCCACTGC |
| *Arg1* | GTGGAGAAAGACATTCCAAGGC | CAGTTCAGGGATCTTGTACCCA |
| *Bdnf* | GGGAAATCTCCTGAGCCGAG | AGCTTTCTCAACGCCTGTCA |
| *Ngf* | GGAGCGCATCGAGTGACTT | CCTCACTGCGGCCAGTATAG |
| *Vgf* | GTCAGACCCATAGCCTCCC | CTCGGACTGAAATCTCGAAGTTC |
| *iNOS* | GGCAGCCTGAGAGACCTTTG | GGAAGCGTTTCGGGATCTGAA |
| *Il-19* | CACACAAGCTCACTTGCACT | GCAAGAATCTGAGAGGCCGA |
| *Il-22* | *GTGCGATCTCTGATGGCTGT* | GACGATGTATGGCTGCTGGA |
| *Aox1* | CATAGGCGGCCAGGAACATT | TCCTCGTTCCAGAATGCAGC |
| *Gpx6* | GTCACGGTTTTGGGCTTTCC | CTGGTGACCGAGTGGAACAA |
| *Ncf1* | *TGGAGGGCAGAGACAATCCA* | AGGGATAGGAGCCGTCTAGG |
| *Vim* | GCAGTATGAAAGCGTGGCTG | CTCCAGGGACTCGTTAGTGC |
| *β-Actin* | CAACGAGCGGTTCCGAT | GCCACAGGTTCCATACCCA |
