# Supplementary material for: Glial cell reactivity and oxidative stress prevention in Alzheimer’s disease mice model by an optimized NMDA receptor antagonist
Source: Sci Rep. 2022 Oct 25;12:17908. doi: 10.1038/s41598-022-22963-x (PMC9596444; doi:10.1038/s41598-022-22963-x)
Supplement: Supplementary file 4 — Supplementary Table 2. [file 41598_2022_22963_MOESM4_ESM.docx]

**Supplementary Table 2.** TRRUST database, analysis for cluster 1, 2 and 3

| **Key TF** | **Description** | **# of overlapped genes** | **P value** | **Q value** | **List of overlapped genes** |
| --- | --- | --- | --- | --- | --- |
| **Nfkb1** | Nuclear factor of kappa light polypeptide gene enhancer in B cells 1, p105 | 5 | 3.95e-06 | 3.42e-05 | *Nos2,Ptgs1,Ccl5,Gclm,Ngb* |
| **Rel** | reticuloendotheliosis oncogene | 3 | 5.26e-06 | 3.42e-05 | *Nos2,Ccl5,Ngb* |
| **Ep300** | E1A binding protein p300 | 3 | 5.36e-05 | 0.000232 | *Txnip,Nos2,Scd1* |
| **Mlxipl** | MLX interacting protein-like | 2 | 0.000151 | 0.00049 | *Txnip,Scd1* |
| **Irf8** | Interferon regulatory factor 8 | 2 | 0.000361 | 0.000797 | *Nos2,Ccl5* |
| **Irf1** | Interferon regulatory factor 1 | 2 | 0.000427 | 0.000797 | *Ccl5,Nos2* |
| **Rela** | v-rel reticuloendotheliosis viral oncogene homolog A (avian) | 3 | 0.000429 | 0.000797 | *Ngb,Ccl5,Nos2* |
| **Hdac1** | Histone deacetylase 1 | 2 | 0.000537 | 0.000873 | *Nos2,Ccl5* |
| **Spi1** | Spleen focus forming virus (SFFV) proviral integration oncogene | 2 | 0.0011 | 0.00159 | *Ccl5,Ncf1* |
| **E2f1** | E2F transcription factor 1 | 2 | 0.00165 | 0.00214 | *Nox4,Nos2* |
| **Fos** | FBJ osteosarcoma oncogene | 2 | 0.00214 | 0.00253 | *Nos2,Ccl5* |
| **Jun** | Jun proto-oncogene | 2 | 0.0112 | 0.0121 | *Ccl5,Nos2* |
| **Sp1** | Trans-acting transcription factor 1 | 2 | 0.0431 | 0.0431 | *Ptgs1,Ngb* |
